# Pharmacokinetics and pharmacodynamics modeling of lonafarnib in patients with chronic hepatitis delta virus infection

**DOI:** 10.1002/hep4.1043

**Published:** 2017-05-19

**Authors:** Laetitia Canini, Christopher Koh, Scott J. Cotler, Susan L. Uprichard, Mark A. Winters, Ma Ai Thanda Han, David E. Kleiner, Ramazan Idilman, Cihan Yurdaydin, Jeffrey S. Glenn, Theo Heller, Harel Dahari

**Affiliations:** ^1^ Program for Experimental and Theoretical Modeling Division of Hepatology, Loyola University Medical Center Maywood IL; ^2^ Centre for Immunity Infection and Evolution, University of Edinburgh Edinburgh United Kingdom; ^3^ Translational Hepatology Unit Liver Diseases Branch, National Institute of Diabetes and Digestive and Kidney Diseases, National Institutes of Health Bethesda MD; ^4^ Department of Medicine Division of Gastroenterology and Hepatology and Department of Microbiology and Immunology, Stanford School of Medicine Stanford CA; ^5^ Laboratory of Pathology National Cancer Institute, National Institutes of Health Bethesda MD; ^6^ Department of Gastroenterology Ankara University Ankara Turkey

## Abstract

The prenylation inhibitor lonafarnib (LNF) is a potent antiviral agent providing a breakthrough for the treatment of hepatitis delta virus (HDV). The current study used a maximum likelihood approach to model LNF pharmacokinetic (PK) and pharmacodynamic (PD) parameters and predict the dose needed to achieve 99% efficacy using data from 12 patients chronically infected with HDV and treated with LNF 100 mg twice daily (bid) (group 1) or 200 mg bid (group 2) for 28 days. The LNF‐PK model predicted average steady‐state LNF concentrations of 860 ng/mL and 1,734 ng/mL in groups 1 and 2, respectively, with an LNF absorption rate k_a_ = 0.43/hour and elimination rate k_e_ = 0.045/hour. The PK/PD model identified an average delay of 0.56 hours and an LNF concentration that decreases HDV production by 50%, EC50 = 227 ng/mL, with a Hill factor *h* = 1.48. The HDV half‐life in blood was 1.87 days, and the average steady‐state LNF efficacy in blocking HDV production was ɛ = 87.7% for group 1 and ɛ = 95.2% for group 2. A biphasic HDV decline with an average phase 1 decline (0.9 log_10_ IU/mL and 1.32 log_10_ IU/mL) was observed in groups 1 and 2, respectively. Phase 2 was not significantly (*P =* 0.94) different between the two groups, with an average slope of –0.06 log IU/mL/day. The model suggests an LNF dose of ∼610 mg bid would achieve ɛ = 99%. *Conclusion*: The first PK/PD modeling study in patients with chronic HDV indicates that a ∼3‐fold increase in LNF dose (∼610 mg bid) would achieve 99% antiviral efficacy. A ritonavir‐boosted LNF combination may provide a means to increase LNF efficacy with minimal side effects. The modeling findings provide an important advance in understanding HDV dynamics and the basis to optimize LNF therapy for hepatitis D. (*Hepatology Communications* 2017;1:288–292)

**Abbreviations**
AUC: area under the curve
bid: twice daily
CDH: chronic delta hepatitis
HBV: hepatitis B virus
HDV: hepatitis delta virus
LNF: lonafarnib
LOWR: Lonafarnib With and Without Ritonavir
NIH: National Institutes of Health
PD: pharmacodynamic
PK: pharmacokinetic
RT‐qPCR: reverse transcription‐quantitative polymerase chain reaction
VK: viral kinetic

## Introduction

An estimated 15 million to 20 million people worldwide are chronically co‐infected with hepatitis delta virus (HDV) and hepatitis B virus (HBV), which results in more severe liver disease progression than chronic HBV mono‐infection.^1^ Unlike the various therapeutic advancements in the field of chronic HBV and hepatitis C virus, there is still no satisfactory or U.S. Food and Drug Administration‐approved therapy for chronic delta hepatitis (CDH).^2^ Guidelines have suggested peginterferon for the treatment of CDH^3^; however, therapy is poorly tolerated and is limited by high relapse rates, even when treatment is extended for 5 years.^4^

A new investigational therapy involves the inhibition of the host function known as prenylation. Prenylation inhibition impacts a vital aspect of the HDV life cycle by disrupting the ability of the viral large delta antigen to interact with the hepatitis B surface antigen, a step that is required to form and secrete infectious HDV particles.^5^ In a recently completed phase 2a dose escalation study, the prenylation inhibitor lonafarnib (LNF) successfully decreased serum HDV‐RNA levels by an average of 0.75 log IU/mL and 1.5 log IU/mL after 28 days of therapy in 100‐mg twice daily (bid) and 200‐mg bid dose groups, respectively.^6^ While LNF has demonstrated potential promise in the therapy of CDH, a better understanding of the dose‐response effect between LNF and HDV could facilitate treatment optimization. Thus, the aim of this study was to estimate the pharmacokinetic (PK), pharmacodynamic (PD), and viral kinetic (VK) parameters during LNF therapy for CDH and to predict the LNF dose needed to achieve 99% efficacy.

## Patients and Methods

### Patients

Fourteen subjects 18 years of age or older were enrolled in a randomized, double‐blinded, placebo‐controlled clinical trial performed at the National Institutes of Health (NIH) Clinical Center (NCT0 1495585). Subjects with CDH as evidenced by the presence of anti‐HDV and quantifiable HDV RNA by reverse transcription‐quantitative polymerase chain reaction (RT‐qPCR) in the serum with compensated liver disease were included. Participants were sequentially assigned into one of two dosing groups. In group 1, 6 patients received 100 mg bid of LNF and 2 patients received placebo. The 2 subjects who received placebo in group 1 were subsequently offered treatment as group 2 participants along with an additional 4 patients who received 200 mg bid of LNF (6 total) and 2 patients who received placebo. Participants received LNF for 28 days and were followed posttherapy for 6 months. Baseline characteristics are shown in http://onlinelibrary.wiley.com/doi/10.1002/hep4.1043/suppinfo. The complete details of this study have been presented elsewhere.^6^ All subjects provided written informed consent. The study was approved according to the Declaration of Helsinki by the Institutional Review Board of the National Institute of Diabetes and Digestive and Kidney Diseases of the NIH.

### DATA COLLECTION

At the start of therapy, subjects were admitted for 72 hours to the NIH Clinical Center for timed blood draws for viral kinetic evaluation, PK, and observation of side effects. While on therapy, subjects underwent predose evaluations on day 7, 21, and 28 in the outpatient clinic. Serum for LNF PK and quantitative HDV RNA were collected and stored at –80°C until the time of analysis.

LNF concentrations in blood were measured by mass spectrometry (QPS, Newark, DE; lower limit of quantification 1.0 ng/mL) before the first dose and at 6, 12, 18, 24, 36, 48, and 72 hours after the first dose. On day 14, intensive PK monitoring was performed in the day hospital for 12 hours as follows: predose, 15 minutes, 30 minutes, 1, 2, 4, 6, and 12 hours postdose. Additional LNF PK samples were measured on days 21 and 28.

Quantitative measurement of serum HDV RNA was performed by RT‐qPCR with a lower limit of detection of 70 IU/mL as described.^6^ Samples were collected before the first dose, at 6, 12, 18, 24, 36, 48, and 72 hours after the first dose and on days 7, 14, 21, and 28.

### MATHEMATICAL MODELING AND PARAMETER ESTIMATION

LNF PK was modeled using a one‐compartment model with lagged first‐order absorption and first‐order elimination (http://onlinelibrary.wiley.com/doi/10.1002/hep4.1043/suppinfo). We modified our HDV‐biphasic model describing viral kinetics^6^ to account for the time‐dependent LNF effectiveness in blocking HDV production, ɛ(t), using the standard E_max_ model (http://onlinelibrary.wiley.com/doi/10.1002/hep4.1043/suppinfo). Model parameters were simultaneously estimated using a maximum‐likelihood method implemented in MONOLIX version 4.2 (http://www.lixoft.com).

### STATISTICAL METHODS

Baseline characteristics (http://onlinelibrary.wiley.com/doi/10.1002/hep4.1043/suppinfo) were compared between dosing groups using the exact Monte Carlo permutation test with 10,000 replications for continuous variables^7^ and Fisher's exact test for categorical covariates, using R 3.1.2. *P <* 0.05 was considered significant.

## Results

### LNF PK

After each intake, LNF concentrations increased to reach a peak (C_max_). In group 1, a median C_max_ = 256 ng/mL (range, 90‐851) was reached after a median time (T_max_) of 4 hours (range, 2.0‐6.0), and the median area under the curve (AUC) for the first 12 hours computed with a trapezoid rule (AUC_0__→__12_) was 1,884 ng‐h/mL (range, 624‐5,592). In group 2, a median C_max_ = 1,073 ng/mL (range, 437‐1,462) was reached at T_max_ = 6 hours (range, 6.0‐12.0), with an AUC_0__→__12h_ = 8,208 ng‐h/mL (range, 2,520‐10,680). LNF PK analysis at steady state is described in the http://onlinelibrary.wiley.com/doi/10.1002/hep4.1043/suppinfo. There was no association between patients' baseline characteristics (http://onlinelibrary.wiley.com/doi/10.1002/hep4.1043/suppinfo) and calculated PK parameters.

### HDV VK

As presented by Koh et al.,^6^ HDV serum viral load decline was biphasic with a first rapid phase and a slower second phase. The absolute viral decline was significantly (*P =* 0.020) greater during phase 1 in group 2 (phase 1 decline, 1.59 log IU/mL) than in group 1 (phase 1 decline, 0.78 log IU/mL). The viral slope during the second phase was not significantly (*P =* 0.94) different between the two groups, with an average slope of –0.06 log IU/mL/day.

### MODEL PREDICTIONS

**Figure 1:**
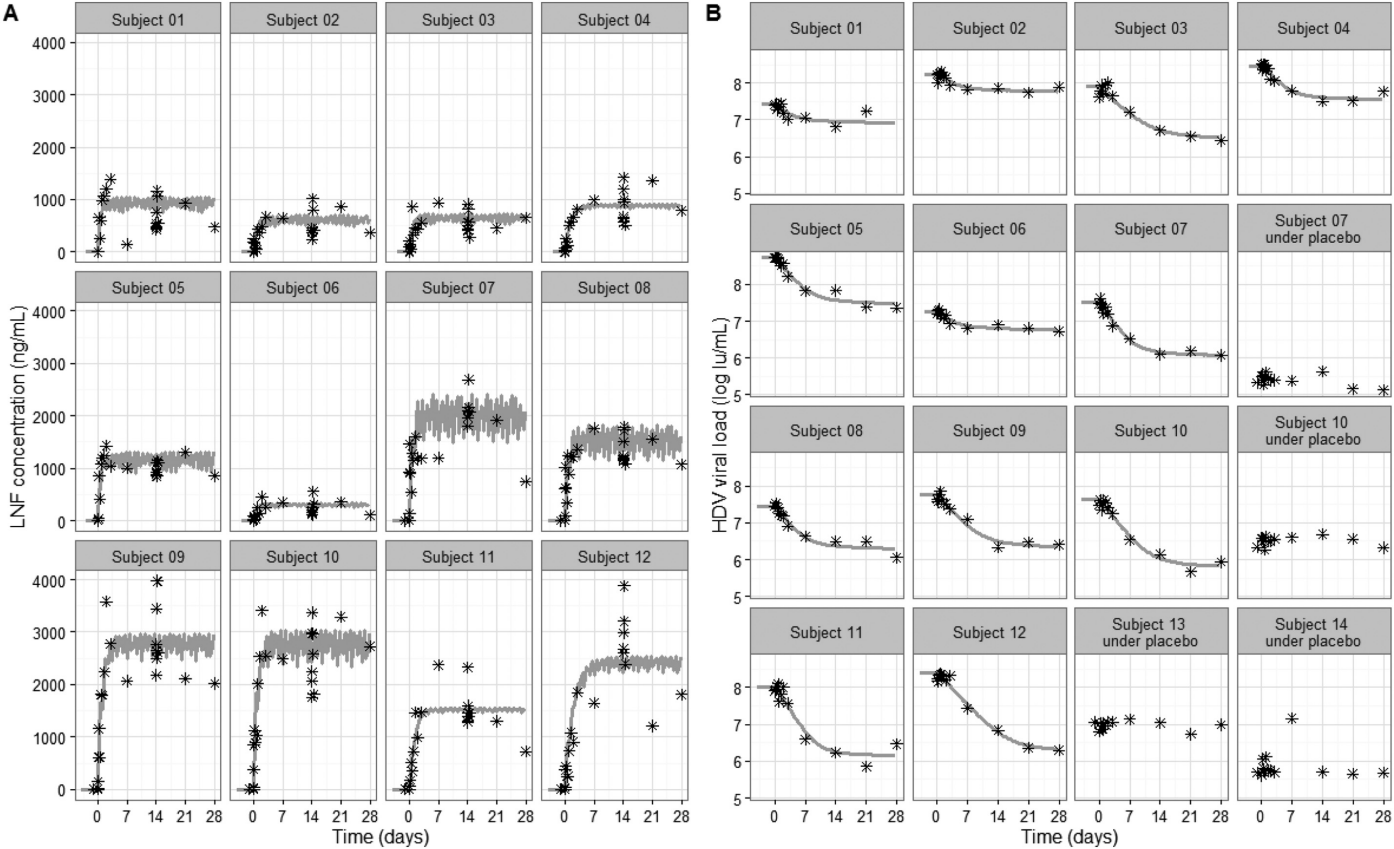
Individual fits of LNF concentration and serum HDV. Each box represents a subject. The measured (A) LNF and (B) HDV RNA levels are shown with stars. Best model fit curves are shown by the gray solid lines. Subjects 01‐06 and subjects 07‐12 were dosed with LNF 100 mg bid and 200 mg bid, respectively. Subjects who received placebo are indicated in (B). Model predictions of LNF PK and HDV response per dosing group are shown in http://onlinelibrary.wiley.com/doi/10.1002/hep4.1043/suppinfo.

The drug absorption rate was estimated as *k_a_* = 0.43/hour, the elimination rate *k_e_* as 0.045/hour (corresponding to an LNF half‐life of 15.4 hours), and a volume of distribution of 212 L. All PK parameters exhibited interindividual variability, ranging from 39% for *k*_e_ to 83% for *k_a_*, and all the fixed‐effect parameters were accurately estimated with relative standard errors <30% (Table 1). The fits are presented in Fig. 1, and the average predictions are presented in http://onlinelibrary.wiley.com/doi/10.1002/hep4.1043/suppinfo. Steady‐state was reached after a 4‐5 half‐life, i.e., 2.6 to 3.2 days. The average steady‐state LNF concentrations were 860 ng/mL and 1,734 ng/mL in groups 1 and 2, respectively. The LNF 50% effective concentration, EC50, was estimated as 227 ng/mL and the Hill factor, *h* = 1.48, translating into an average steady‐state drug efficacy ɛ of 87.7% and 95.2% for groups 1 and 2, respectively. Our model predicts that in order to achieve an increase of LNF efficacy from 95% to 99% in blocking HDV production, an average steady‐state LNF concentration of 5,063 ng/mL would be required, corresponding to an average monotherapy LNF dose of ∼610 mg bid.

**Table 1:** PK/PD/VK PARAMETER ESTIMATIONS

Parameter [unit]	Parameter Description	Population Estimate (rse %)	Interpatient Variability % (rse %)
t_0_ [hr]	Pharmacological delay	0.56 (16)	52 (23)
ka [hr^–1^]	LNF absorption rate	0.43 (28)	83 (27)
Vd/F [L]	Effective volume of distribution	223 (15)	49 (23)
ke [hr^–1^]	Elimination rate	0.045 (13)	39 (25)
Emax	Maximal effectiveness	1.0 (1)	‐**
EC50 [ng/mL]	LNF concentration leading to 50% of effectiveness	227 (26)	62 (23)
h	Hill factor	1.48 (7)	‐
V_0_ [log10 IU/mL]	Pretreatment HDV viral load	7.88 (2)	5.7 (21)
δ [d^–1^]	Infected cell loss rate	0.01 (FIXED)*	‐
c [d^–1^]	Free virus clearance rate	0.37 (10)	21 (48)

* The death/loss rate of productively HDV‐infected cell was fixed to δ = 0.01/day as described.^6^ In nonlinear mixed effect models the population estimate is described by the fixed effect and the interindividual variability by the random effect. ** Fitting the model with no interindividual variability for *Emax*, *h,* and *δ* provided the best fits; this implies that *Emax*, *h,* and *δ* have the same value for all subjects.

Abbreviation: rse, relative standard error.

HDV viral kinetics was well captured by the model (Fig. 1). Overall, HDV kinetic parameter estimates were consistent with the previous analysis,^6^ with an HDV clearance rate of 0.37/day, corresponding to a virus half‐life of 1.87 days and an initial viral load of 7.88 log_10_ IU/mL. The interindividual variability for these parameters was relatively small and was estimated as 21% for the virus clearance rate and 6% for the initial viral load.

## Discussion

LNF concentrations observed here in patients with chronic HDV were comparable to those observed in a dose escalation study of LNF for treatment of patients with solid tumors who received 25 mg to 300 mg bid^8^ and after a single dose (100 mg) in healthy volunteers.^9^ Moreover, C_max_, T_max_, and AUC_0__→__12h_ values computed in the current study were within the range observed during day 1 and day 14 of treatment in Castaneda et al.^8^ using 100 and 200 mg bid of LNF. The available data suggest that LNF PK features are not altered in patients with chronic liver disease, such as HBV/HDV infection, compared to patients without liver disease.

The mathematical modeling in the current study predicts that LNF might achieve 99% efficacy in blocking HDV production for concentrations above 5,063 ng/mL, corresponding to an LNF dose of ∼610 mg bid (i.e., ∼3‐fold higher than administered in group 2). However, since we recently reported that an LNF dose of 200 mg bid was associated with poor gastrointestinal tolerability,^6^ higher monotherapy LNF doses may not be a feasible therapeutic approach. Interestingly, HDV kinetic data from a subsequent phase 2 study has shown encouraging results with LNF 100 mg bid plus ritonavir.^10^ Ritonavir boosting provides a potential means to decrease LNF doses in the gastrointestinal tract, thereby limiting adverse effects, while increasing its postabsorbed concentration and antiviral efficacy. Specifically, in the phase 2 LOna farnib With and without Ritonavir (LOWR) HDV‐1 study, the serum LNF concentrations with LNF 100 mg bid plus ritonavir 100 mg once a day were up to 6,000 ng/mL compared to ∼900 ng/mL with LNF 100 mg bid (clinical trial: NCT02430181). The LNF concentrations observed with ritonavir boosting in LOWR HDV‐1 exceeded the above‐predicted 99% efficacy concentration and were associated with dramatic HDV viral load declines and better tolerability than higher doses of LNF monotherapy (Yurdaydin et al., manuscript submitted). Ritonovir boosting of LNF is being further evaluated in the phase 2 LOWR HDV‐2 study (clinical trial: NCT02511431).

In conclusion, the current study presents the first PK and PD characterization of LNF for hepatitis D infection. The model fits well to both measured LNF PK and HDV kinetics observed in 12 patients infected with CDH who were treated with 100 or 200 mg bid for 28 days. While a projected LNF dose of 610 mg bid would achieve 99% efficacy, adverse effects limit the use of high doses of LNF. The mathematical modeling provides a strong rationale for ritonavir boosting as a potential strategy to optimize the antiviral effectiveness and the tolerability of lonafarnib.

Author names in bold designate shared co‐first authorship.

## Supporting information

Additional Supporting Information may be found at http://onlinelibrary.wiley.com/doi/10.1002/hep4.1043/suppinfo.

Supporting Information
