# Supplementary material for: Pharmacokinetics and pharmacodynamics modeling of lonafarnib in patients with chronic hepatitis delta virus infection
Source: Hepatol Commun. 2017 May 19;1(4):288–92. doi: 10.1002/hep4.1043 (PMC5721397; doi:10.1002/hep4.1043)

**Appendix**

Table A1. Subjects’ baseline characteristics

| **Patient Demographics** | Group 1 (N = 6) | Group 2 (N = 6) | p-value |
| --- | --- | --- | --- |
| Male – no. (%)* | 5 (83) | 4 (67) | 1.0 |
| Median Age-Yr (IQR) | 36 (30-42) | 45 (38-55) | 0.36 |
| Median BMI (IQR) | 22.8 (21.9-23.5) | 26.1 (25.5-27.3) | 0.51 |
| Pre-Tx Nucleoside Analogues (%)* | 2 (33) | 2 (33) | 0.75 |
| Race - no. (%)* |  |  | 0.77 |
| Asian | 3 (50) | 3 (50) | 1.0 |
| Caucasian | 3 (50) | 3 (50) | 1.0 |
| African | 0 | 0 | 1.0 |
| **Disease Characteristics** |  |  |  |
| Median Ishak Fibrosis (IQR) | 3 (3, 3.5) | 3 (3, 3.75) | 0.78 |
| Median ALT U/L (IQR) | 125 (86-168) | 63 (52-162) | 0.40 |
| Median AST U/L (IQR) | 61 (50-75) | 42 (41-60) | 0.54 |
| Median Platelet K/uL (IQR) | 174(170-183) | 197 (180-229) | 0.48 |
| Median Total Bilirubin mg/dL (IQR) g/dL(IQR) | 0.35 (0.30-0.48) | 0.4 (0.40-0.60) | 1.0 |
| Median log HDV RNA IU/mL (IQR) | 6.06 (5.56-6.60) | 5.92 (5.62-6.26) | 0.93 |
| Median HBV DNA IU/mL (IQR) | 463 (<21-1239) | 635 (<21-947.5) | 0.60 |
| Median HBsAg ng/mL (IQR) | 17707 (11268-32085) | 11417 (8970-13103) | 0.67 |

***** Effect of LNF dosing group was tested with Fisher’s exact test for categorical variables. For continuous variables, the association was tested with exact Monte Carlo permutation test.

**PK model**

For a 1-compartment model with lagged 1^st^ order absorption and a 1^st^ order elimination, the serum concentration, C(t), is described by^(11)^:

$$C\left( t \right)=\left\{ \begin{aligned} 0 if t-t_{Di}\leq Tlag \\ \sum_{i=1}^{n} \frac{F.D_{i}.ka}{Vd(ka-ke)}\left( e^{-ke\left( t-t_{Di}-Tlag \right)}-e^{-ka\left( t-t_{Di}-Tlag \right)} \right) else \end{aligned} \right.$$

Where

F: bioavailability

D_i_: Dose at the i^th^ intake included in the model as a regressor

t_Di:_ time of dose i

ka: absorption rate

ke: elimination rate

Vd: volume of distribution

Tlag: lag-time

i (from 1 to n): number of dose

The contribution of each i^th^ dose is assumed to be 0 between t_Di_ and t_Di_+Tlag.

This leads to an average steady-state concentration of:$Css=\frac{F.D}{ke.Vd.\tau}$, where τ is the time interval between doses. Therefore one can compute the dose corresponding to a given Css as $D=\frac{Css.ke.Vd.\tau}{F}$

**LNF PK at steady state**

At steady-state, computed from the frequent measures at day 14, a median C_max_ of 1075 ng/mL (range: 559-1416) was reached at T_max_ of 4 hours after dosing (range: 2.0 – 6.0) and the AUC_0🡪12_ was 8760 ng-h/mL (range: 4056-12216) in Group 1. In Group 2, a median C_max_ of 3031 ng/mL (range: 1786-3974) was reached at T_max_ of 5 hours after dosing (range: 0.3 – 6.0) with an AUC_0🡪12_ of 22944 ng-h/mL (range: 17496-40656).

**Prediction of the dose needed to reach 99% effectiveness**

Using the population parameters, the steady state effectiveness is given by $\varepsilon_{ss}=E_{max}\frac{{Css}^{h}}{{Css}^{h}+{EC}_{50}^{h}}$. We can therefore compute the steady state concentration, Css, leading to ε_ss_ = 0.99 as $C_{ss}=\sqrt[h]{\frac{.99}{Emax-.99}}{EC}_{50}$.

Since $Css=\frac{F.D}{ke.Vd.\tau}$, we can then compute the dose needed to reach steady state effectiveness of 99% $D_{99}=\frac{\sqrt[h]{\frac{.99}{Emax-.99}}{EC}_{50}.ke.Vd.\tau}{F}$.

**The Emax model**

$$\varepsilon\left( t \right)=E_{max}\frac{{C(t)}^{h}}{{C(t)}^{h}+{EC}_{50}^{h}}$$

Where E_max_ is the maximum effectiveness, the constant EC_50_ is the LNF concentration at which the drug’s effectiveness in blocking viral production is half its maximum, *h* is the Hill coefficient, which determines how steeply the effectiveness rises with increasing concentration, and C(t) the LNF serum concentration.

**Biphasic Model**

$\frac{dI}{dt}=\beta VT_{0}-\delta I$

$\frac{dV}{dt}=p\left( 1-\varepsilon(t) \right)I-cV$

Here *I* represents infected cells that can produce HDV virions, and *V*, is the HDV RNA level in blood. Similar to a previous modeling effort^(6)^, we assume the target cell count was constant during 28 days with LNF treatment and equal to its pre-treatment steady-state value *T_0_* = *cδ/βp*. Free HDV infects target cells with rate constant *β*, generating infected cells, *I*, which produce new HDV virions at rate *p* per infected cell. Infected cells are lost at a rate *δ* per infected cell. HDV is assumed to be cleared from blood at rate *c* per virion. Lonafarnib is considered to block viral production in time-dependent factor (1-*ε(t)*), where *ε(t)* is the Emax model (above) which is defined as the effectiveness of drug in preventing viral production/secretion, with a value *ε* = 1, implying 100% effectiveness.

**Parameters estimation**

We simultaneously fitted LNF concentrations and HDV RNA data using a non-linear mixed effect (nlme) model. In nlme models, the fixed effect describes the population parameters and the random effect reflects the inter-individual variability (IIV). Model parameters were estimated using a maximum-likelihood method implemented in MONOLIX version 4.2 (<http://software.monolix.org>). Due to the limited number of data points during the second phase decline, we met identifiability issues regarding parameter δ. We therefore fixed δ to 0.01 d^-1^, as previously done^(6)^. Additionally, since *F* is not known and cannot be identified from the available data, we redefined *Vd* as *Vd/F*, which is the effective volume of distribution, estimated in Table 1.

**Figure A1. Population predictions** for LNF concentration (upper left), viral load (upper right), effectiveness as 1-log_10_(1-ε) (lower left) for Group 1 (100 mg bid) in grey, Group 2 (200 mg bid) in black and 610 mg bid in blue (i.e. the predicted dose inducing an effectiveness of 99%).


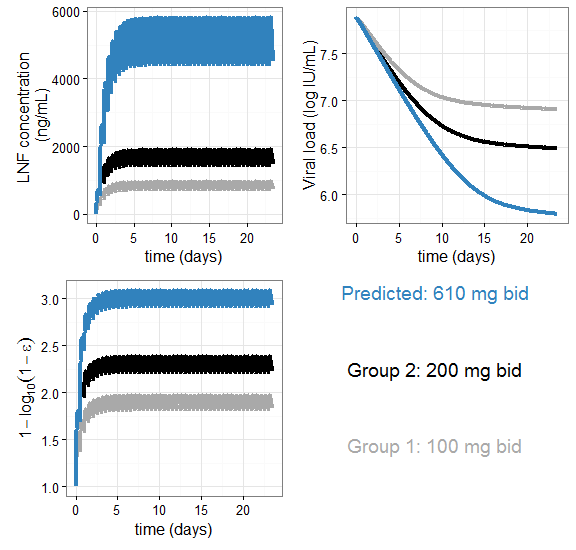


**Figure A2. Magnified view of Figure 1.** The model captures the main kinetic features of LNF concentrations and fits HDV viral kinetics during 28 days of therapy. The frequent measurements of LNF concentration at day 14 show large fluctuations that could not be fully captured by the model in all subjects.


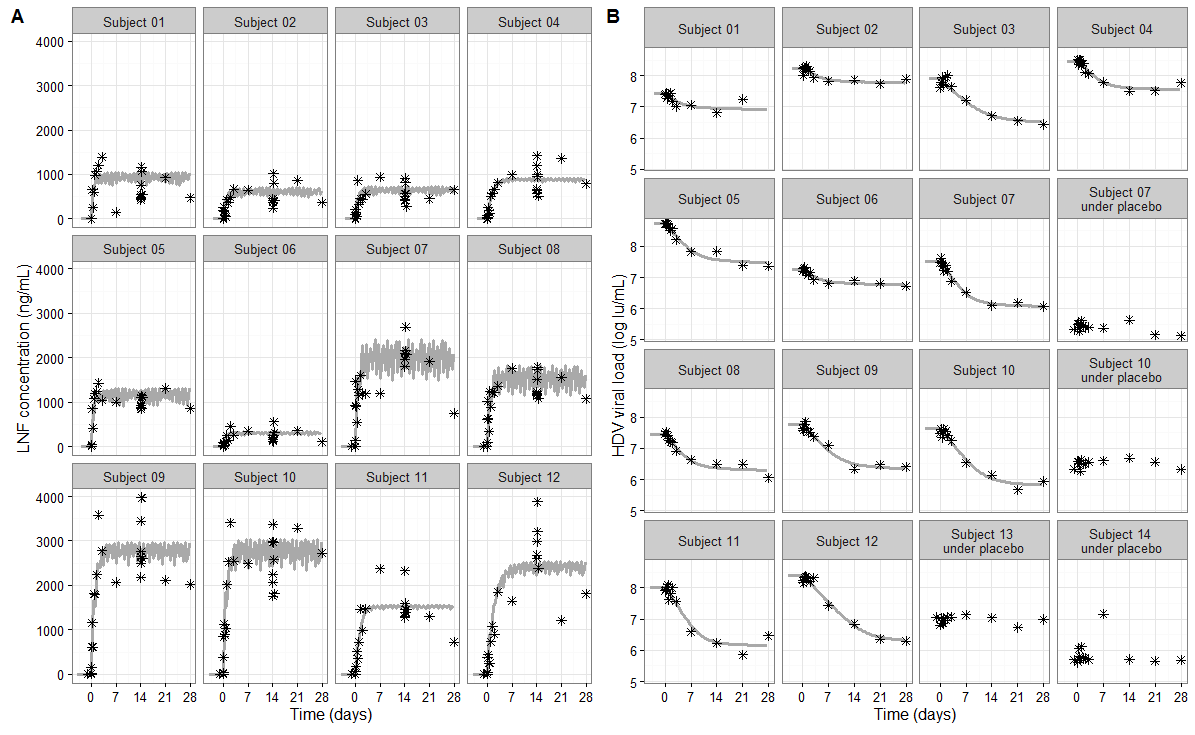

Supplement: Supplementary file 1 — Supporting Information [file HEP4-1-288-s001.docx]
